# Expression and Characterization of the *Spats1* Gene and Its Response to E2/MT Treatment in the Chinese Soft-Shelled Turtle (*Pelodiscus sinensis*)

**DOI:** 10.3390/ani12141858

**Published:** 2022-07-21

**Authors:** Luo Lei, Junxian Zhu, Chen Chen, Yakun Wang, Xiaoyou Hong, Xiaoli Liu, Lingyun Yu, Chengqing Wei, Haigang Chen, Yihui Liu, Ruiyang Li, Wei Li, Xinping Zhu

**Affiliations:** 1 Key Laboratory of Tropical and Subtropical Fishery Resource Application and Cultivation, Ministry of Agriculture and Rural Affairs, Pearl River Fisheries Research Institute, Chinese Academy of Fishery Sciences, Guangzhou 510380, China; 2019213005@stu.njau.edu.cn (L.L.); zhujunxian_1994@163.com (J.Z.); chenchen@prfri.ac.cn (C.C.); wangyk@prfri.ac.cn (Y.W.); hxy@prfri.ac.cn (X.H.); liuxl@prfri.ac.cn (X.L.); yuly@prfri.ac.cn (L.Y.); zjweichengqing@prfri.ac.cn (C.W.); zjchenhaigang@prfri.ac.cn (H.C.); ll9412152022@163.com (Y.L.); a3021893023@163.com (R.L.); 2 Wuxi Fisheries College, Nanjing Agricultural University, Wuxi 214081, China; 3 College of Fisheries and Life Science, Shanghai Ocean University, Shanghai 201306, China

**Keywords:** cloning, expression, *Pelodiscus sinensis*, *Spats1*, steroid hormone

## Abstract

**Simple Summary:**

Spermatogenesis in *Pelodiscus sinensis* has distinct seasonal characteristics. *Spats1* acts a key function in germ cell differentiation in mammals, yet its study in *P. sinensis* has not been reported. Here, we cloned the coding sequences (CDS) of *Spats1* for the first time and characterized its expression in different tissues. In addition, the expression characteristics of *Spats1* in the testis during different seasons and the expression pattern in embryos and adults after steroid hormone treatment were also investigated. The present research provides a preliminary study on the physiological regulation of *Spats1* in *P. sinensis*, and lays the foundation for further systematic investigation of the molecular mechanisms of spermatogenesis and release in *P. sinensis*.

**Abstract:**

*Spats1* (spermatogenesis-associated, serinerich 1) has been characterized as a male-biased gene which acts an important role in the germ cell differentiation of mammals. Nevertheless, the function of *Spats1* in the Chinese soft-shelled turtle (*P. sinensis*) has not yet been reported. To initially explore the expression of *Spats1* in *P. sinensis* and its response to sex steroid treatment, we cloned the CDS of *Spats1* for the first time and analyzed its expression profile in different tissues, including the testes in different seasons. The *Spats1* cDNA fragment is 1201 base pairs (bp) in length and contains an open reading frame (ORF) of 849 bp, which codes for 283 amino acids. *Spats1* mRNA was highly expressed in the testes (*p* < 0.01) and barely detectable in other tissues. In *P. sinensis*, the relative expression of *Spats1* also responsive to seasonal changes in testis development. In summer (July) and autumn (October), *Spats1* gene expression was significantly higher in the testes than in other seasons (*p* < 0.05). *Spats1* mRNA was found to be specifically expressed in germ cells by chemical in situ hybridization (CISH), and it was mainly located in primary spermatocytes (Sc1), secondary spermatocytes (Sc2) and spermatozoa (St). *Spats1* expression in embryos was not significantly changed after 17α-methyltestosterone (MT)and 17β-estradiol (E2) treatment. In adults, MT significantly induced *Spats1* expression in male *P. sinensis*. However, the expression of *Spats1* in testes was not responsive to E2 treatment. In addition, the expression of *Spats1* in females was not affected by either MT or E2 treatment. These results imply that *Spats1* is a male-specific expressed gene that is mainly regulated by MT and is closely linked to spermatogenesis and release in *P. sinensis.*

## 1. Introduction

Spermatogenesis is a complex physiological process that mainly includes spermatogonia cell differentiation and sperm formation [1]. Spermatogenesis occurs continuously in majority of mammals and birds without any relation to the season. However, spermatogenesis in reptiles is closely associated with the seasons [2], such as in wall lizard (*Podarcis muralis*) [3], Asian yellow pond turtles (*Mauremys mutica*) [4], and tropical rattlesnake (*Crotalus durissus*) [5]. *P. sinensis* is an important species of aquaculture which is broadly spread in Asia. Since the fast growth of turtle farming, the economic value of *P. sinensis* has also increased [6]. Phylogenetic analysis revealed that *P. sinensis* is a relatively ancient tetrapod, and the study of the morphological evolution and reproductive patterns of the species is of great scientific value [7,8]. In *P. sinensis*, spermatogenesis is a seasonally dependent physiological process which starts in the spring and proceeds through the summer and fall. Spermatogenesis occurs in May, and mature sperm is released at the end of October [9,10]. From December to the following March, *P. sinensis* enters hibernation, wherein sperm production is inactive [11]. Currently, studies on spermatogenesis of *P. sinensis* are mostly carried out by a histological examination [12], while less research has been conducted at the molecular level.

Sex hormones normally act as essential regulators in gonadal differentiation and sexual activity. In many species, the direction of sexual differentiation and the expression patterns of some genes related to sexual activity are modified by the treatment of sex steroid hormones [13,14]. In *P. sinensis*, sex steroid hormone treatment leads to sex reversal and affects the expression patterns of some genes that are closely related to sex differentiation. For example, significant masculinization of genetic female embryos and feminization of genetic male embryos were observed following aromatase inhibitor (AI) and E2 treatment. E2 could significantly downregulate the mRNA and protein expressions of doublesex-and-mab-3-related transcription factor 1 (*Dmrt1*) and SRY-box transcription factor 9 (*Sox9*), while AI exerted the opposite effect [15]. Although the trend of *R-spondin 1* (*Rspo1*) expression was inconsistent across different periods of embryonic development, it was up- and down-regulated after exogenous estradiol and letrozole treatment, respectively [16].

Spermatogenesis is a complex process that involves a multiplicity of growth factors and cytokines [17]. *Spats1* (spermatogenesis-associated serine-rich 1) is a gene that is tightly associated with spermatogenesis in mammals [18]. It was established that *Spats1* is an evolutionarily conserved, testis-specific protein which is differentially expressed during rat male meiotic prophase [19], and the highest level of *Spats1* expression was detected in primary spermatocytes among the different spermatogenic cell types [20]. Furthermore, *Spats1* has been shown to be a highly phosphorylatable protein [21]. In addition, recent studies have identified *Spats1* as a new molecular target in colorectal cancer [22]. However, no reports on the *Spats1* in *P. sinensis* have been found.

Of interest, according to transcriptome data (Accession: PRJNA838782) of ovary and testis in the early development of *P. sinensis* [23], *Spats1* was characterized as a male-specific expressed gene. In the present study, we obtained cDNA fragments of the *Spats1* in *P. sinensis*, and explored the tissue distribution of *Spats1* and seasonal patterns in testes for the first time. Significantly, this study also explored for the first time the response of *Spats1* to E2 and MT treatment in the embryonic and adult gonads of *P. sinensis*. These findings contribute to the clarification of the physiological functions of *Spats1* in *P. sinensis,* providing new viewpoints to investigate the regulatory mechanism of *Spats1* on spermatogenesis in *P. sinensis*, and enriching the basic data for *Spats1* in reptiles.

## 2. Materials and Methods

### 2.1. Animals and Ethics Statement

All experimental *P. sinensis* adults and embryos were obtained from Caixing Industrial Co. (Huizhou, China). All experimental procedures were performed in accordance with the regulations for animal care of the Pearl River Fisheries Research Institute (Guangzhou, China).

### 2.2. Collection of Samples

#### 2.2.1. Cloning and Tissue Distribution Expression

A total of six *P. sinensis* were used for cloning and tissue distribution expression. Healthy three-year-old *P. sinensis* females and males (*n* = 3 each) were anesthetized with an intraperitoneal injection of 0.05% MS-222 (20 mg/kg, Sigma, St. Louis, MO, USA), and sacrificed. The heart, liver, spleen, kidney, brain, muscle, ovary and testis were sampled. Subsequently, these tissues were washed slightly with 1× PBS in diethylpyrocarbonate (DEPC) water, and stored in liquid nitrogen for total RNA extractions.

#### 2.2.2. Seasonal Expression and In Situ Hybridization

A total of 36 male *P*. *sinensis* were used for exploring *Spats1* expression in different seasons and localization in cells. Three healthy *P*. *sinensis* males ranging from 1 to 2 and 3-years-old were acquired each time in January, April, July, and October. Animals were anesthetized using 0.05% MS-222 (Sigma, St. Louis, MO, USA), and testes were collected and stored in liquid nitrogen for total RNA extractions. Another portion of the testis tissue was placed in 4% paraformaldehyde (PFA) at 4 °C for overnight fixation. Subsequently, it was dehydrated using a methanol gradient and finally kept in 100% methanol at −20 °C until use.

#### 2.2.3. Hormone Treatment in Embryonic Development

A total of 300 fertilized eggs were collected, identified, and put into the incubator in the laboratory of the Pearl River Fisheries Research Institute during 12 h after spawning. According to the reasonable temperature range of incubation, referring to the previous studies [24,25,26,27], the incubation temperature was kept at 31 °C, and the humidity was held at 75% in this study. These 300 fertilized eggs were divided into three groups: the MT-treated group, the E2-treated group, and the control group. Referring to the research of Cai et al. [28], embryos were treated with E2 and MT. E2 and MT were solubilized in 95% ethanol at a concentration of 20 μg/μL, and 5 μL of the medication was applied to the eggshell topically in the adjacent areas of embryonic development at stages 15 and 16 (gonadal differentiation usually starts at Stage 17) [29]. The control group was processed with 5 μL of 95% ethanol. After hormone treatment, incubation conditions in the treatment and control groups remained at a temperature of 31 °C and 75% humidity. At stage 25, the gonads were rapidly isolated from the embryo (both kidneys and gonads) and then frozen and stored in liquid nitrogen for RNA extraction. DNA from other tissues was extracted with a kit (Mabio, Xiamen, China) and the gender of the embryos was determined through PCR amplification (primer ZHB1) [30].

#### 2.2.4. Hormone Treatment in Adults

A total of 106 adult *P. sinensis* were used for hormone treatment, of which 53 were females and 53 were males. The gonads of three males and three females were randomly collected as a blank control group before hormone treatment. The remaining 50 females *P. sinensis* were equally divided into 2 groups (E2 treatment group, MT treatment group), and the remaining 50 males were also equally divided into 2 groups (E2 treatment group, MT treatment group), and injected with E2 and MT from the leg muscles at a concentration of 50 μg/μL and a dose of 10 mg/kg. The gonads of three females and three males were randomly collected from each group at 0 h, 6 h, 12 h, 24 h, 48 h, and 7 days after hormone treatment and placed in liquid nitrogen for total RNA extractions.

### 2.3. RNA Extraction and cDNA Synthesis

Total RNA from different tissues was extracted by RNA iso Plus (Takara, Beijing, China) according to the instructions. The quality of RNA was detected according to RNA electrophoresis (Bio-Rad, PowerPacTM, CA, USA) and NanoDrop 2000 Spectrophotometer (Thermo Fisher, NanoDropOne, MA, USA). Total RNA from different tissues was used as the template and the first-strand cDNA was synthesized following the protocol of the reverse transcription kit (Takara, Beijing, China).

### 2.4. Cloning and Sequencing

The primers (PsSpats1-1F/PsSpats1-1R) (Table 1) for *Spats1* fragment amplification were designed based on RNA-Seq. The *Spats1* was amplified with Taq Enzyme PCR (Accurate Biology, Changsha, China) according to the instructions. This step used approximately 2 μg of cDNA from the testes of *P. sinensis* with the primers PsSpats1-1F and PsSpats1-1R. The PCR conditions were 94 °C for 30 s, 35 cycles of 98 °C for 10 s, 58 °C for 30 s, 72 °C for 60 s, and 72 °C for 2 min, followed by cooling to 4 °C. Products were electrophoresed in 1.5% agarose gels. The target fragments were purified using a Universal DNA Purification Kit (TIANGEN, Beijing, China), and then ligated with the pMD19-T vector (Takara, China). Afterwards, the products were transformed into *E. coli* DH5α competent cells following the manual, and the recombinants were screened by blue–white color on LB plates containing ampicillin. Positive clones were identified by PCR. The PCR conditions were 94 °C for 30 s, 35 cycles of 98 °C for 10 s, 58 °C for 30 s, 72 °C for 60 s, and 72 °C for 2 min. The sequences of all product were verified at Tianyi Huiyuan (Guangzhou, China).

### 2.5. Structural Analysis

All amino acid sequences were obtained from NCBI GenBank and analyzed by DNAMAN 6.0. The segmentation of signal peptides is predicted with the SignalP-5.0 server program (http://www.cbs.dtu.dk//services/SignalP/index.php accessed on 6 June 2022). ClustalX 1.83 was used to perform amino acid multiple sequence alignment and the protein 3D model was derived by SWISS-MODEL server. A phylogenetic tree was modeled by maximum likelihood (ML) method of MEGA 7 and ClustalW (https://myhits.sib.swiss/cgi-bin/clustalw accessed on 6 June 2022) through amino acid sequences.

### 2.6. Cryostat Sections of the Gonad

The testes were collected in July from the three ages (1, 2, and 3 years) and fixed overnight at 4 °C in 4% PFA. The testes were immersed in 30% sugar-PBS buffer at 4 °C for 5 h after PBS washing. Samples were wrapped with O.T.C. (Tissue-Tek. Sakura Finetek USA, Inc., Torrance, CA, USA) and cut into 4-μm sections using a cryostat microtome (Leica, Wetzlar, Germany). The tissue sections were placed on slides and subsequently stored at −80 °C. Frozen tissue sections are mounted on frosted glass slides (4951 PLUS-001E, Thermo Fisher, MA, USA) and held at −80 °C till the next step of the experiment.

### 2.7. In Situ Hybridization

The amplification product gained from primers (PsISH Spats1-3F and PsISH Spats1-3R) (Table 1) designed based on the *P. sinensis Spats1* cDNA sequence were ligated to the plasmid pMD19-T (Takara, Beijing, China). Plasmid extraction was performed on positive clones. The vector plasmid was linearized by restriction enzymes Apa I and Sac II, and precipitated overnight by addition of ethanol and sodium acetate. Wash the precipitate with 75% ethanol and subsequently solubilize it using DEPC water. Antisense and sense probes were synthesized with the digoxigenin RNA Labeling Kit (Roche, Basle, Switzerland), which uses T7 and Sp6 promoters. In situ hybridization was carried out following the protocol of the Reagent Kits (G3024) (Servicebio, Wuhan, China). BCIP/NBT chromogenic solution was used in Chemical in situ hybridization (CISH). Propidium iodide (PI) was used to stain nuclei. The sections were mounted with an anti-quenching mounting agent (Gold Antifade reagent, Invitrogen, Shanghai, China). Observations and photographs were taken using an Axio Observer Z1 microscope and an Axiocam 506 color imaging system (ZEISS, Oberkochen, Germany).

### 2.8. Semi-Quantitative PCR and Real-Time Quantitative PCR

Based on the *Spats1* cDNA sequence of *P. sinensis,* a pair of specific primers PsSpats1-2F and PsSpats1-2R were designed for the semi-quantitative PCR performed (Table 1). The PCR condition was 35 cycles at 98 °C for 10 s, 58 °C for 30 s, and 72 °C for 60 s. The primer (*Eef1a*-F/*Eef1a*-R) (Table 1) was used as a control with a PCR amplification fragment of 252 bp. Separate amplification products with 1.5% agarose gel, stained with ethidium bromide, and photographed with the Alpha Innotech bioimaging system.

The qRT-PCR was performed using the same primers (PsSpats1-2F and PsSpats1-2R) (Table 1), and *Eef1a* was used as the internal reference gene. Follow the instructions of iTaq Universal SYBR Supermix (BIO-RAD, CA, USA) for qRT-PCR. The PCR volume was 20 μL, containing SYBR Supermix (10 μL), cDNA (1 μL, 100 ng), each primer (1 μL, 2 μM), and nuclease-free water (7 μL). Following PCR conditions were applied for all primers: 95 °C for 10 min; 40 cycles of 95 °C for 15 s, 55–60 °C for 15 s, and 72 °C for 15 s; melting curve analysis occurred at 95 °C for 15 s, 60 °C for 60 s, and 95 °C for 15 s. Three replicate experiments were analyzed for each sample. The expression level of *Spats1* was normalized with the expression of *Eef1a*, then analyzed by the 2^−∆Ct^ method for the expression level of *Spats1*.

### 2.9. Statistical Analysis

Normality tests were first performed to ensure that the *t*-test was applicable to the data analysis of this experiment, followed by Student’s *t*-test or one-way ANOVA using SPSS 25.0 software (SPSS Inc., Chicago, IL, USA). All values are presented as a mean ± SEM. *p* < 0.05 was considered statistically significant.

## 3. Results

### 3.1. Cloning and Sequence Analyses

**Figure 1:**
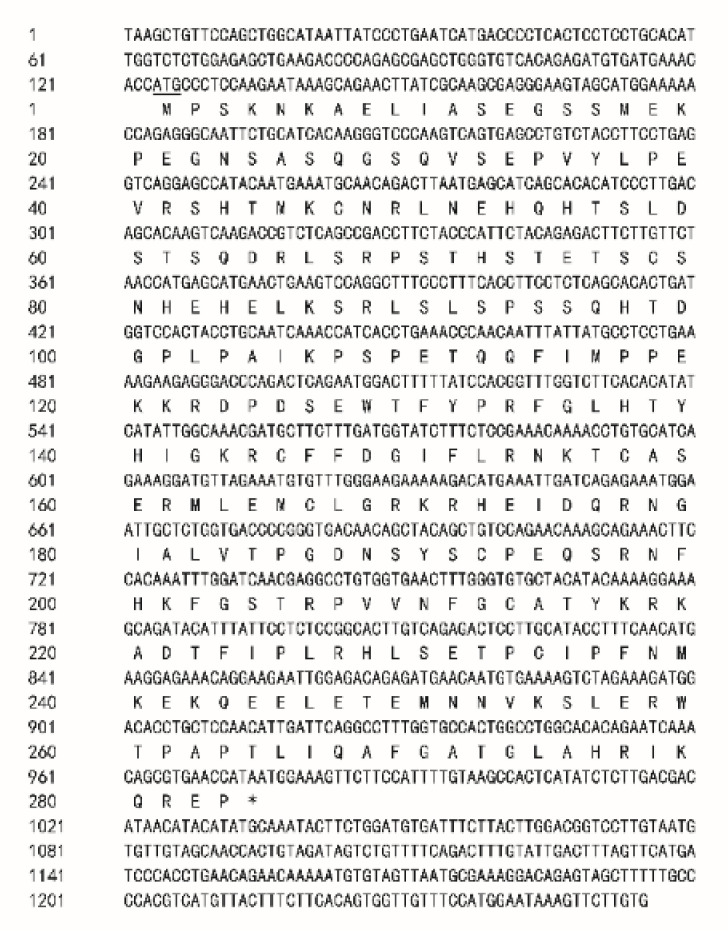
Nucleotide and predicted amino acid sequences of *P*. *sinensis Spats1.* The start codon (ATG) is underlined by a single line, and the stop codon (TAA) is represented by the asterisks.

The *Spats1* cDNA fragment is 1201 base pairs (bp) in length (Figure 1) and contains an open reading frame (ORF) of 849 bp, which encodes for a protein with 283 amino acids.

**Figure 2:**
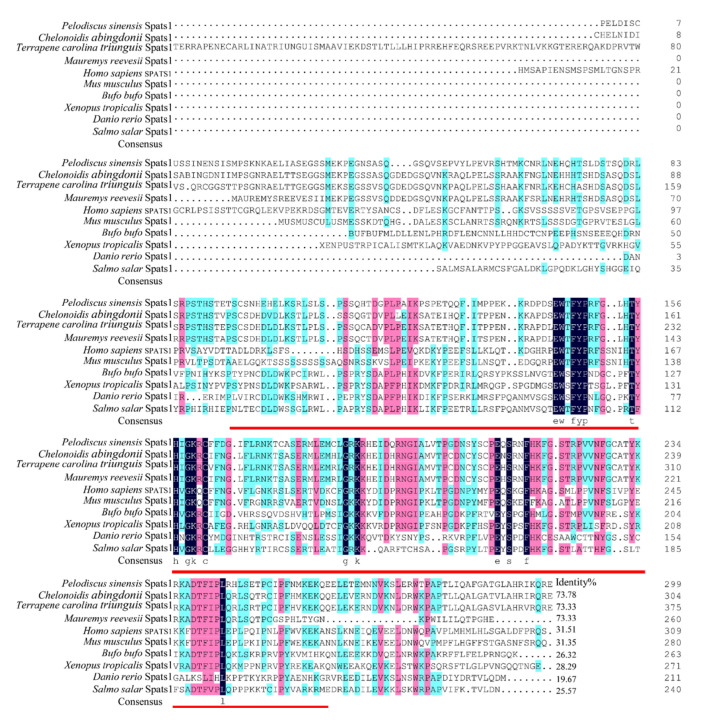
Alignment of the amino acid sequences. The structural domain is underlined in red. GenBank accession numbers: *Chelonoidis abingdonii* Spats1 (XP_032655998.1), *Terrapene carolina triunguis* Spats1 (XP_029767743.1), *Mauremys reevesii* Spats1 (XP_039386593.1), *Homo sapiens* SPATS1 (NP_001359010.1), *Bufo bufo* Spats1 (XP_040286913.1), *Danio rerio* Spats1 (XP_017208226.1), *Mus musculus* Spats1 (NP_081925.2), *Xenopus tropicalis* Spats1 (XP_002936034.2), and *Salmo salar* Spats1 (XP_014000180.1). Black shading indicates the same amino acid sequence. Pink shading indicates conserved sequences with more than 75% of the listed polypeptides. The blue shading indicates the conserved sequence with more than half of the listed peptides.

The inferred molecular weight of this protein is 32.15 kDa, and shares a 73.78% identity with *Chelonoidis abingdonii* Spats1 (XP_032655998.1), 73.33% with *Terrapene carolina triunguis* Spats1 (XP_029767743.1), and 73.33% with *Mauremys reevesii* Spats1 (XP_039386593.1). Yet, the sequence had low homology with those of mammals, fish, and amphibians, such as *Homo sapiens* SPATS1 (NP_001359010.1) (45.08%), *Bufo bufo* Spats1 (XP_040286913.1) (26.32%), and *Danio rerio* Spats1 (XP_017208226.1) (19.67%) (Figure 2).

**Figure 3:**
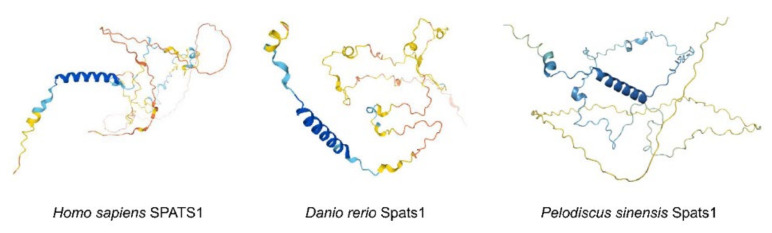
The protein 3D structure models. Different colors represent different model confdence.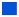: Very high (Plddt > 90);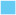: Confident (90 > Plddt > 70);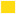: Low (70 > Plddt > 50);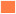: Very low (Plddt < 50).

**Figure 4:**
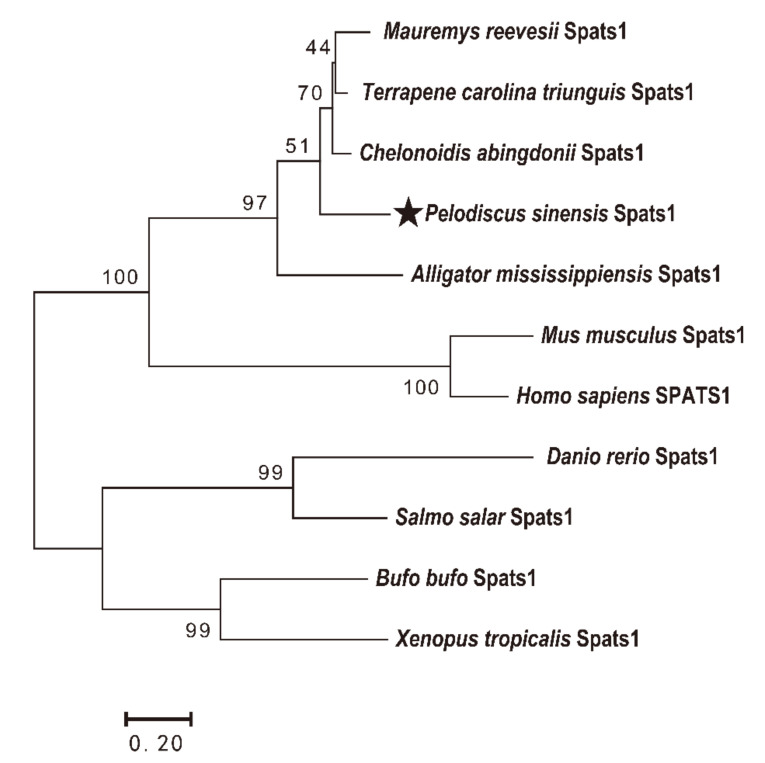
Phylogenetic tree constructed through the maximum likelihood method. The scale bar shows the genetic distance. The number at each node indicates the bootstrap value obtained for 1000 replicates.

The protein 3D structures of human (SPATS1) and zebrafish (Spats1) are similar in that they both begin with two α-helices at the C-terminus and are followed by a certain number of shorter α-helices interconnected by disordered loops. Although the N-terminal part of Spats1 in *P*. *sinensis* also starts with two α-helices, the number of subsequent short α-helices is smaller than that in both human and zebrafish (Figure 3). To better illustrate the evolutionary relationships of the *Spats1* gene between different species, a phylogenetic tree was structured by maximum likelihood (ML) from the putative amino acid sequences of 11 species, inclusive of *P*. *sinensis*. The Spats1 proteins were classified into four clusters: mammalia, reptilia, amphibia, and teleosts; these clades were strongly supported by the bootstrap values (Figure 4). *P*. *sinensis* Spats1 was classified in the reptile cluster and was associated more strongly with Spats1 from turtles than from other species.

### 3.2. Tissue Distribution

**Figure 5:**
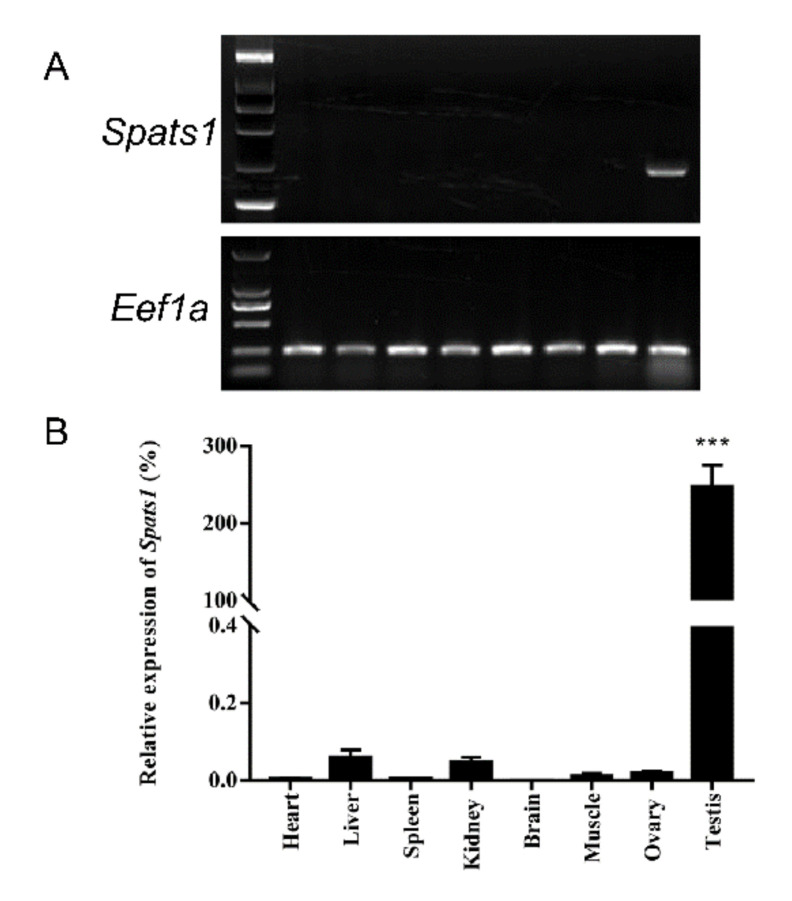
The distribution of *Spats1* mRNA in different tissues. (**A**) Tissue distribution gel electrophoresis map. (**B**) Tissue distribution assay results using qRT-PCR. *** *p* < 0.001.

Tissue distributions of *Spats1* in three-year-old *P. sinensis* specimens were analyzed by Semi-Quantitative PCR and qRT-PCR, using *Eef1a* as a reference gene. Gel electrophoresis maps showed bright amplified band of *Spats1* only in testis (Figure 5A). *Spats1* was detected to highly expressed in testes via qRT-PCR (DF = 13, F = 682.449, *p <* 0.001), but rarely found in other tissues (Figure 5B). Hence, it was demonstrated that *Spats1* mRNA was sex-specific expressed in the gonads and was only in the adult testes of *P. sinensis*.

### 3.3. The Cellular Distribution of Spats1 mRNA in Testes

**Figure 6:**
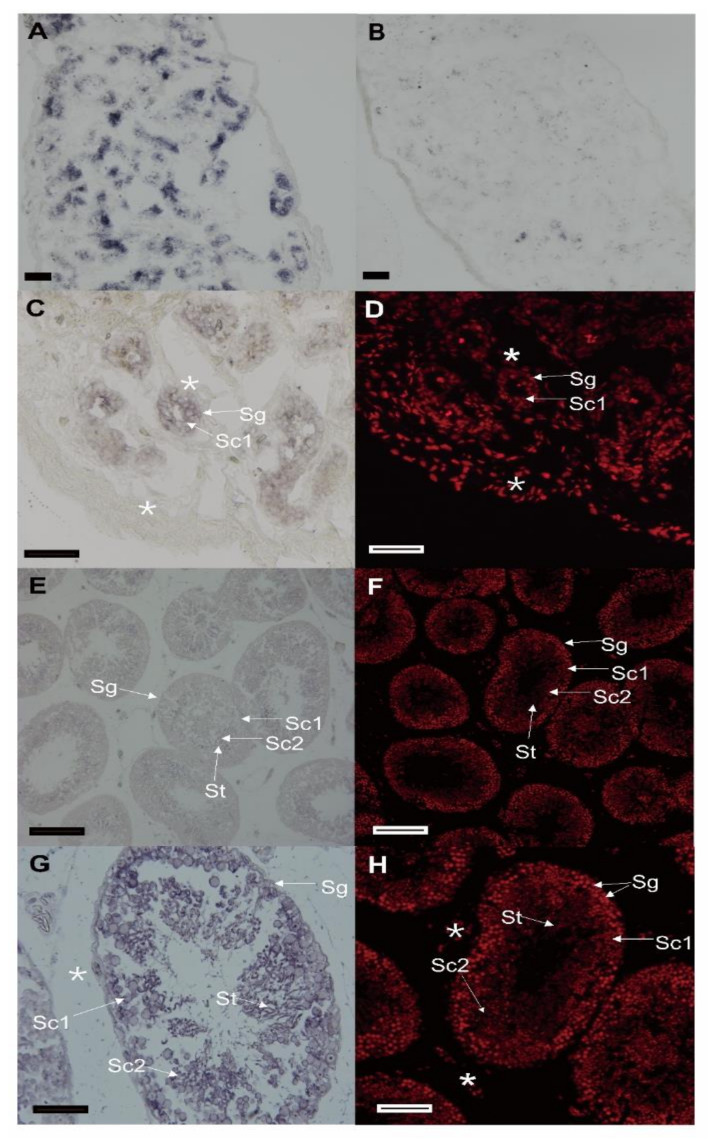
Localization of *Spats1* mRNA in testes by chemical in situ hybridization (CISH). The signals were developed by alkaline phosphatase (AP) staining (purple). *: somatic cells, St: spermatozoa, Sc1: primary spermatocytes, Sc2: secondary spermatocytes, Sg: spermatogonia. (**A**) Detected signal by antisense probe in testis section. (**B**) No signal was detected by sense probe in testis section. (**C**,**D**) The testis sections of one-year-old specimen; (**C**) Localization of *Spats1* in different cells; (**D**) Nucleus was stained with propidium iodide (PI). (**E**,**F**) The testis sections of a two-year-old specimen; (**E**) Localization of *Spats1* in different cells; (**F**) Nucleus was stained with PI. (**G**,**H**) The testis sections of a three-year-old specimen; (**G**) Localization of *Spats1* in different cells. H: Nucleus was stained with PI. Scale bars = 50 µm.

This is the first time that *Spats1* mRNA was localized in the male germ cells of 1-, 2-, and three-year-old *P. sinensis* specimens. The CISH of the testis sections revealed that chemical signals of *Spats1* transcripts were detected with the antisense probes (Figure 6A,C,E,G), while no signal was observed with sense probes (Figure 6B). The nucleus was stained with PI (Figure 6D,F,H). *Spats1* mRNA was mainly distributed in primary spermatocytes (Sc1), secondary spermatocytes (Sc2), and spermatozoa (St). However, in spermatogonia (Sg) and somatic cells (*), *Spats1* expression was weakly (Figure 6A,C,E,G).

### 3.4. Expression Pattern of Spats1 mRNA in Testes of Different Seasons

**Figure 7:**
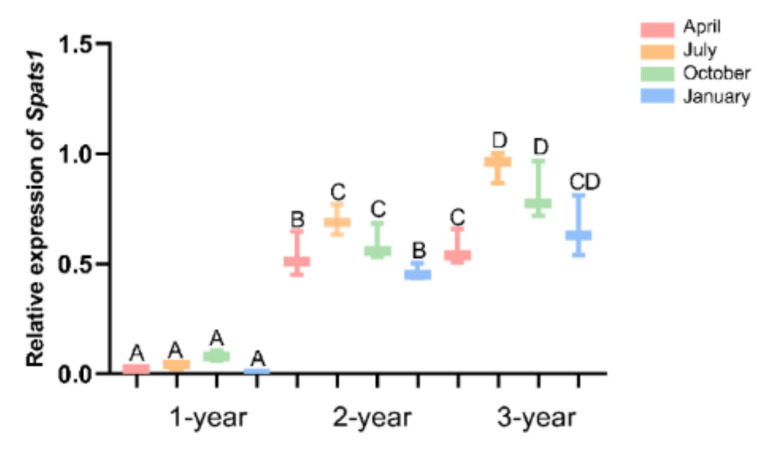
Expression of *Spats1* in testes of different ages in different seasons. Different letters represent the significance of differences between different groups (*p* < 0.01).

In *P. sinensis*, spermatogenesis is accompanied by a distinct periodic rhythm. In this study, the expression pattern of the sex-bias gene *Spats1* in *P. sinensis* testes was studied. qRT-PCR was used to test the relative expression levels of *Spats1* mRNA in testes at different seasons. Overall, the expression of *Spats1* in male *P. sinensis* appeared to increase with age. The expression of *Spats1* was significantly lower in one-year-old males than in two- and three-year-old males in different seasons (DF = 24, F = 15.048, *p* < 0.01). Moreover, the expression of *Spats1* in one-year-old male was not significantly different between seasons. In two- and three-year-old males, the expression pattern of *Spats1* was significantly different across seasons (DF = 24, F = 15.048, *p* < 0.01). *Spats1* expression was lowest in April and highest in July (DF = 24, F = 15.048, *p* < 0.01). *Spats1* expression gradually decreased after July, but *Spats1* expression was still significantly higher in October than in April (DF = 24, F = 15.048, *p* < 0.01) (Figure 7).

### 3.5. Effect of E2 and MT Treatment on Embryos

**Figure 8:**
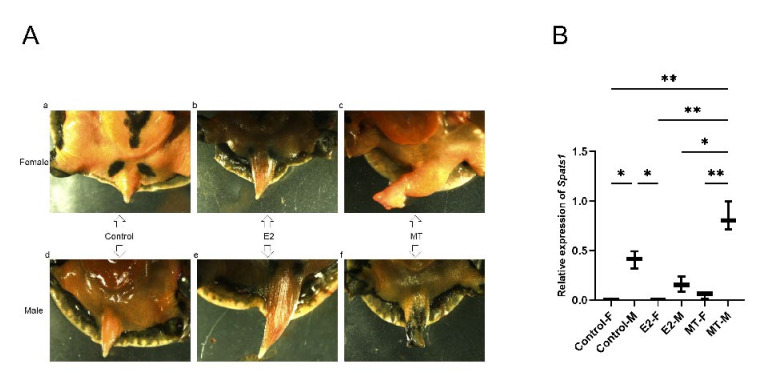
(**A**) E2- and MT-treated embryonic tails. (**a**) Female controls; (**b**) E2-treated females; (**c**) MT-treated females; (**d**) Male controls; (**e**) E2-treated males; (**f**) MT-treated males. (**B**) *Spats1* mRNA expression. Control-F: Control Female; Control-M: Control Male; E2-F: E2-treated females; E2-M: E2-treated males; MT-F: MT-treated females; MT-M: MT-treated males; Significant differences are represented by asterisks (* *p* < 0.05, ** *p* <0.01).

Compared to the untreated control, the tails of the newly hatched male and female *P. sinensis* turtles became more elongated after the E2 treatment (Figure 8A(b,e)). In contrast, the tails of MT-treated male and female *P. sinensis* became shorter and stouter (Figure 8A(c,f)). The qRT-PCR results showed that *Spats1* expression was decreased in embryonic testes after E2 treatment and up-regulated after MT treatment compared to the control group, but none of the differences were significant. In addition, neither E2 nor MT treatment had a significant effect on *Spats1* expression in female embryos (Figure 8B).

### 3.6. E2 and MT Treatment Were Performed on Adult Male and Female P. sinensis

**Figure 9:**
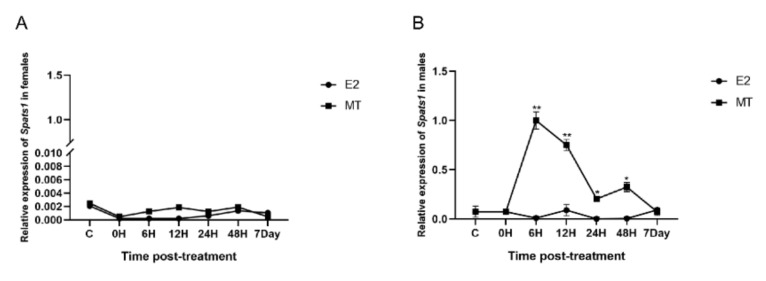
Relative expression of *Spats1* gene in adults after E2 and MT treatment. (**A**) Effects of E2 and MT on transcript abundance of *Spats1* gene in female adult *P. sinensis.* (**B**) Effects of E2 and MT on transcript abundance of *Spats1* gene in male adult *P. sinensis*. * means significant differences (*p* < 0.05); ** means significant differences (*p* < 0.01).

E2 and MT treatments were performed on adult male and female *P. sinensis*, and it was found that both E2 and MT treatments could barely significantly affect *Spats1* expression in female. At 6 h, 12 h, 24 h, 48 h, and even 7 days after E2 and MT treatment, *Spats1* expression in females was always weak (Figure 9A). In contrast, in males, the expression of *Sptas1* in testes increased significantly after MT treatment, peaked at 6 h, and then began to gradually decrease. However, at 12 h, its expression level remained significantly higher than that of the untreated controls (DF = 14, F = 60.518, *p* < 0.01). 24 and 48 h after MT treatment, the expression level of *Spats1* in the testes continued to decrease, but was still significantly higher than the untreated controls (DF = 14, F = 60.518, *p* < 0.05). It was not until day 7 that the expression level of *Spats1* decreased to the same level as the initial expression. Interestingly, the expression of *Spats1* in the testes was almost unchanged after E2 treatment, either at 6 h, 12 h, 24 h, 48 h, or after 7 days (Figure 9B).

## 4. Discussion

In this study, the *Spats1* cDNA was cloned and characterized. An 849 bp cDNA fragment of *Spats1* encoding 283 amino acids was obtained. For protein 3D structures, although the N-terminal portion of Spats1 in *P. sinensis* begins with two α-helices, as in zebrafish and humans, the number of subsequent short α-helices is smaller than it is in either. The Spats1 3D structure in *P. sinensis* differs significantly from that of zebrafish and humans, suggesting that in *P. sinensis* may be evolutionarily more distant from humans and zebrafish. The results of protein 3D structures and multiple comparative analyses indicated the extensive homology of the *P. sinensis Spats1* gene with Testudinidae animals. This implies that the protein function of Spats1 in turtles may be different from that of other species.

Of the tissues examined in this study, *Spats1* mRNA expression was highest in the testis. *Spats1* has shown a similar expression profile in various tissues in mice [20]. Interestingly, the distribution of *Spats1* in different tissues was almost consistent with the pattern of *Dmrt1* and anti-Müllerian hormone (Amh), which have been shown to be closely associated with testis differentiation in *P. sinensis* [15,31]. Similar to the gonadal structure of other vertebrates, the essential functional unit for sperm production in the testes of *P. sinensis* is the seminiferous tubule [32]. The arrangement of cells from the spermatogenic epithelium to the lumen is somatic cells, spermatogonia, primary spermatocytes, secondary spermatocytes, round spermatids, and sperm [33,34,35]. To obtain insight into the function of *Spats1* in the development and differentiation of male germ cells, cellular localization of its transcripts in the testis was investigated. The obtained results revealed that *Spats1* mRNA was mainly located in spermatocytes, secondary spermatocytes, and spermatozoa. In rats, *Spats1* attains maximum levels during meiosis of the first spermatogenic wave, mostly in pachytene spermatocytes, whereas the signal was weaker in spermatogonia, Sertoli cells, and myoid cells [20]. Despite the fact that Spats1 is a testis-specific protein, the Spats1 loss-of-function mouse model analysis revealed that knockdown of Spats1 did not affect fertility in either males or females [36]. The testis-specific expression of *Spats1* and the expression pattern in different germ cells imply that *Spats1* may be closely associated with testicular differentiation and male germ cell differentiation in *P. sinensis*. In view of the differences in spermatogenesis mechanisms between reptiles and mammals, it remains to be investigated whether *Spats1* has the same effects on fertility in *P. sinensis* as in rats.

Seasonally breeding animals undergoes a periodic fluctuation in gonad development. In male reptiles, spermatogenesis and steroidogenesis have a highly seasonality [37,38,39,40]. In this study, *Spats1* expression in one-year-old males was very weak throughout the year, and *Spats1* expression was highest in the testes at three years old. Testosterone expression increased with age, and the peak of testosterone occurred at the peak of spermatogenic activity [41,42]. This implies that the differential expression of *Spats1* in different ages may be closely associated with the expression of testosterone. In addition, spermatogenesis of *P. sinensis* is initiated in the summer (June), and the mature spermatozoa are subsequently released from the epididymis in the autumn (October) [6,43]. In *P. sinensis*, the seminiferous epithelium is spermatogenically active in summer and autumn, but quiescent during the remaining part of the year [43]. The expression pattern of *Spats1* in this experiment implies that *Spats1* expression is seasonally influenced and closely related to spermatogenesis and release. Interestingly, a recent report on *P. sinensis* mentioned that the expression pattern of high-mobility group box 2 (Hmgb2) in the testes also strongly correlates with the season [44]. Therefore, we speculated that *Spats1* regulates sperm production and release and might be affected by effective accumulative temperature [45].

Gonadal development and sexual activity are not only determined by the interactions of multiple genes but also by environmental factors, such as temperature or hormone exposure [46,47], and exposure to exogenous hormones during gonadal differentiation can alter the population gender ratio [48,49]. Steroid hormones are natural and induce sex change processes [50], among which E2 is one of the most potent estrogens impacting sex differentiation [51] and MT is one of the most potent androgens impacting sex differentiation [52]. For instance, E2 induces sex reversal in *P. sinensis* and the protandrous barramundi (*Lates calcarifer*) [53,54], and MT controls secondary sexual characteristics in *Siniperca chuatsi* as well [55]. The same phenomenon has been confirmed in mammals and fish [56]. *P. sinensis* embryo is an excellent model for studying gonadal development and reproduction since development takes place in the egg, and we can intervene directly *in vitro*. In this experiment, both male and female *P. sinensis* embryonic tails changed significantly after E2 and MT treatments compared to the untreated group, indicating that the sexual characteristics of *P. sinensis* were affected by E2 and MT. The treatment of embryos with hormones resulted in significant alterations in the expression of many genes [57,58]. The expression of some sexually dimorphic genes is significantly altered in the embryo after sex hormone treatment in *P. sinensis*, such as *Dmrt1*, *Sox9* [15], and cytochrome p450 family 19 subfamily a (Cyp19a) [59]. However, in the present experiment, the expression of *Spats1* in embryos was not significantly altered after E2 and MT treatment. In contrast, in adults, *Spats1* expression was significantly increased in males after MT treatment, which is similar to the pattern in zebrafish, where the expression of gonadotrophin-releasing hormones 2 and 3 (*gnrh2* and *gnrh3*) is upregulated after MT treatment [57]. The expression pattern of *Spats1* in embryos and adults after sex hormone treatment suggests that *Spats1* responds to sex endosteroid hormone regulation only in adults, and it is hypothesized that the gene may not be involved in sex differentiation in *P. sinensis*. Androgenic stimulation results not only in the production of seminal fluid, but also promotes the maturation of sperms [60]. In this experiment, the significant elevation of *Spats1* expression after MT treated in adults, implying that *Spats1* may be positively regulated by MT, which in turn promotes sperm maturation and release in *P. sinensis*. Studies have shown that estrogen is important for sperm activity and that estrogen treatment affects the expression of sex-related genes [61,62]. Yet, the expression of *Spats1* in male *P. sinensis* changed weakly after E2 treatment in this experiment. This is different from the response pattern of other sexually dimorphic genes in adult *P. sinensis* after E2 and MT treatment, such as forkhead box L2 (*Foxl2*), whose expression was significantly altered in both males and females after either E2 or MT treatment [63]. This might be related to the concentration of estrogen treatment or that *Spats1* is not located in the estrogen regulatory signaling pathway in *P. sinensis*. As for the fact that *Spats1* expression in females has been very weak and almost unaffected by E2 and MT treatments, there are two possible reasons: inadequate concentration of hormone treatment, or the fact that *Spats1* is not involved in female physiological activities at all. Thus, the specific regulation mechanism involved in this process needs to be further studied.

## 5. Conclusions

In summary, *Spats1* is specifically expressed in the testis of *P. sinensis* with a seasonal expression pattern and is involved in spermatogenesis and release. Moreover, E2 and MT treatments revealed that *Spats1* only responded to MT treatment both in embryos and in adults. Thus, additional research is necessary to investigate the issue in the future. This study enriches the data on *Spats1* in *P. sinensis* and provides a reference for future studies on the molecular mechanisms of reproductive activity in this species and even other reptiles.

## Figures and Tables

**Table 1:** Primers used in experiments.

Name	Application	Sequences (5′—3′)	Amplification Efficiency	Product Size (bp)
PsSpats1-1F	Clone	CCCTGAATCATGACCCCTCA		1201
PsSpats1-1R	Clone	TCCATGGAAACAACCACTGTGAA		
PsSpats1-2F	Semi-Quantitative/qRT-PCR	AGCGAGGGAAGTAGCAT	98.25%	251
Ps Spats1-2R	Semi-Quantitative/qRT-PCR	GGAAGGTGAAAGGGAAAA		
Ps Spats1-3F	CISH	TAATACGACTCACTATAG GGCGAGCCGACCTTCTACCCA		869
Ps Spats1-3R	CISH	ATTTAGGTGACACTATAGAA TACTCTCTGTCCTTTCGCATTA		
*Eef1a*-F	Semi-Quantitative/qRT-PCR	ACTCGTCCAACTGACAAGCCTC	100.73%	337
*Eef1a*-R	Semi-Quantitative/qRT-PCR	CACGGCGAACATCTTTCACAG		
ZHB1-F	Sex identification PCR	CTGGAAACAATATCATCGCCGAG		590
ZHB1-R	Sex identification PCR	TGTGTGCCGTGCCTGCGA		

## Data Availability

All datasets generated or analyzed during this study are included in the published article.
